# Enhancing the Shelf Life and Microbial Safety of Refrigerated Eggs Using Edible Coatings of Aloe Vera Gel Combined With Fennel and Lime Peel Essential Oils

**DOI:** 10.1002/fsn3.72389

**Published:** 2026-09-27

**Authors:** Zahra Ranjbarinasab, Fatemeh Shahdadi, Maryam Yousefi, Zohreh Seyedimarghaki, Shima Omidi

**Affiliations:** ^1^ Department of Animal Science, Faculty of Agriculture University of Jiroft Kerman Iran; ^2^ Department of Food Science and Technology, Faculty of Agriculture University of Jiroft Kerman Iran; ^3^ Department of Food Science and Engineering Islamic Azad University, Shahreza Branch Shahreza Iran

**Keywords:** aloe vera gel, egg, essential oil, food safety, Haugh unit, natural preservatives, weight loss

## Abstract

In order to investigate the effect of coating with aloe vera gel (100%), different levels of fennel and lime peel essential oil (0, 500, and 1000 ppm) on some qualitative and microbial characteristics of eggs at refrigerated temperature (5°C) during three storage periods (1, 15, and 30 days), an experiment was conducted as a factorial in a completely randomized design with three replications. During the storage period, the weight loss (%), Haugh unit, shell thickness, albumen and yolk pH, albumen foam production and total bacterial count were investigated. The results showed that the highest weight after 30 days of storage, the highest weight loss was observed in the control (4.81%), whereas all coated treatments showed significantly lower weight loss (3.35%–3.36%). The highest Haugh unit values were recorded in the treatments coated with aloe vera gel containing 1000 ppm fennel essential oil (65.74) and 1000 ppm lime peel essential oil (65.40), whereas the lowest value was observed in the control (54.17). The highest pH of egg white and yolk was observed in the control compared to other experimental treatments on days 15 and 30. Shell thickness was not affected by the experimental treatments (*p* > 0.05). At the end of the storage, the highest total microbial count was observed in the control (5.40 log CFU/g) and the lowest in the aloe vera + 1000 ppm fennel essential oil (4.16 log CFU/g) and aloe vera + 1000 ppm lime peel essential oil (4.17 log CFU/g) treatments. In general, it can be concluded that coating with aloe vera gel along with fennel and lime peel essential oil, particularly at 1000 ppm, can be used as an effective combination to reduce the microbial load and maintain the quality of the internal contents of eggs during the storage period.

## Graphical abstract

This study investigated the effectiveness of an edible coating based on aloe vera gel (100%) combined with fennel and lime peel essential oils for improving the quality and microbial safety of refrigerated eggs. During refrigerated storage, application of the coating reduced egg weight loss, helped maintain a higher Haugh unit, moderated changes in albumen and yolk pH, improved albumen foam production, and reduced the total microbial count.

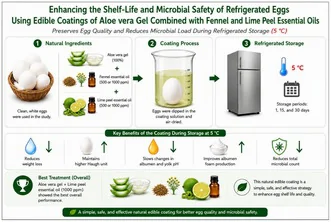

## 1 Introduction

Eggs are essential in the diet of most people due to their high protein and valuable nutrients. The popularity of eggs is related to their nutritional value. The water content of eggs is approximately 74.4% and their protein and fat content are 12.3% and 11.6%, respectively (Sharaf Eddin et al. 2019). Eggs are a rich source of digestible fats such as choline, unsaturated fats, cholesterol, and phospholipids rich in cephalin, which, in addition to maintaining the structural integrity of cell membranes, are also essential for the growth of nerve cells. Eggs are usually considered a low‐energy protein source. Approximately 148 kcal is obtained from 100 g of eggs (Mann et al. 2008). Eggs are also a rich source of lysine and sulfur‐containing amino acids (Zaheer 2015). In addition, eggs are rich in essential vitamins and minerals, making them one of the most nutritionally complete animal‐derived foods (Antonino et al. 2024).

However, eggs are susceptible to physical and microbial spoilage and can rapidly lose quality due to the loss of moisture and carbon dioxide through the nearly 10,000 tiny pores. These pores in the eggshell also facilitate the penetration of certain microorganisms into the egg and contaminate its internal contents (Nys et al. 2011). This damage can be prevented by coating. An edible coating is defined as a thin (edible) layer of food material that forms directly on the surface of the food (Ananey‐Obiri et al. 2018). Such coatings prevent the penetration of microorganisms into the eggshell. As a result, they increase the shelf life of eggs and reduce economic losses. Previous studies have concluded that coatings help maintain internal quality, increase shell strength, and reduce the microbial load on the shell surface (Falguera et al. 2011). Recent studies have confirmed that natural biopolymer‐based edible coatings effectively improve egg quality and microbial safety during storage (Cortés‐Ramírez et al. 2024).

Nowadays, the use of plant materials such as gels, extracts and essential oils for coating food products has received attention. Aloe vera ( *Aloe barbadensis* Miller) is an herbaceous plant of high therapeutic importance that has been widely investigated for its application in food, pharmaceutical and cosmetic industries (Maan et al. 2018). Aloe vera gel is composed of soft and viscous tissues containing parenchyma cells. It is a transparent gel‐like substance with a complex composition and contains a variety of bioactive compounds consisting of carbohydrates, proteins, fibers, soluble sugars, vitamins, minerals, amino acids, organic acids, and phenolic compounds (Sharrif Moghaddasi and Res 2011; Maan et al. 2018). In the food industry, Aloe vera gel is used in the preparation of functional foods, as a natural preservative or as a material for edible films/coatings (Maan et al. 2018). Over the past decades, it has been considered as an edible film/coating due to its effectiveness in increasing the shelf life of food products. The antioxidant and antimicrobial potential of aloe vera gel films/coatings makes them an important example of natural and active packaging. Recent advances have shown that incorporating bioactive compounds into biopolymer matrices can enhance the functionality of edible coatings. For example, Yang et al. (2026) and Tayebi Rad et al. (2026) demonstrated the effectiveness of natural bioactive compounds in improving food preservation and active packaging performance.

Essential oils are used as natural antimicrobial agents for food preservation (Napiórkowska et al. 2024). Lime peel essential oil is a mixture of more than 100 compounds, divided into two volatile parts (99%–85% of the total essential oil) and the non‐volatile part (1%–15%). The volatile parts include monoterpenes (such as limonene), sesquiterpene hydrocarbons and oxygenated derivatives [aldehydes (such as citral), ketones and acids (along with linear aldehydes), alcohols (such as linalool)] and hydrocarbons, fatty acids, sterols, carotenoids, waxes, coumarins, and flavonoids (Paw et al. 2020). In general, lime peel essential oils have potent antioxidant and antibacterial properties (Raspo et al. 2020). Fennel, scientifically known as *Foeniculum vulgare* L., is a plant of the *Apiaceae* family that is cultivated worldwide for its aromatic seeds and leaves and is rich in essential oil (up to 5%) (Marčac et al. 2023). Fennel essential oil is a clear, pale‐yellow liquid, and its main components include trans‐anisole (about 65% to 78%). Fennel essential oil also contains alpha‐pinene, terpene, geraniol, alpha‐furanene, and limonene, which have good bacteriostatic effects against *E. coli* , *Bacillus subtilis* , and *Staphylococcus aureus* (Santos et al. 2019; Chang et al. 2016). Recent studies have further confirmed the effectiveness of essential oil‐enriched edible coatings in extending the shelf life of perishable foods (Antonino et al. 2024). In a study, the effect of rice protein coatings enriched with tea tree, copaiba ( *Copaifera langsdorffii* ), and thyme essential oils on quality of fresh eggs was evaluated. The results showed that use of coatings based on rice protein concentrate enriched with different essential oils influences the internal quality of eggs during storage and may be an effective alternative for increasing the shelf life of commercial eggs (Pires et al. 2020). Al Awwaly et al. (2024) investigated the effect of casein‐chitosan edible coating with garlic essential oil on chicken characteristics egg. The result showed that chicken eggs coated with chitosan casein edible coating with the addition of garlic essential oil 1% for storage time of 0 day, 7 days, 14 days, 21 days, and 28 days gave a very significant effect on egg yolk pH, egg albumen pH, Haugh unit and egg yolk index.

Despite these advances, limited information is available regarding the combined application of Aloe vera gel with fennel and lime peel essential oils for preserving table eggs. To the best of our knowledge, no previous study has investigated this combination for maintaining both the physicochemical quality and microbial stability of refrigerated eggs. Therefore, the aim of the present study was to investigate the effect of edible coatings containing aloe vera gel with fennel and lime peel essential oils on improving the shelf life and quality of eggs.

## 2 Materials and Methods

The chemicals used included sodium sulfate, ethanol, and agar plate culture medium (Merck Company, Germany).

### 2.1 Preparation of Fennel and Lime Peel Essential Oils

The required amount of dried Mexican lime peel ( *Citrus aurantifolia* ) was dried at room temperature and in the shade. The essential oils of ground lime peel and fennel seed powder were separately extracted by steam distillation using a Clevenger‐type apparatus for 3 h according to the European Pharmacopeia guidelines (Benítez et al. 2013). The resulting essential oils, after filtration and dehydration with anhydrous sodium sulfate, were stored in sealed dark glass containers at 5°C until use.

### 2.2 Preparation of Aloe Vera Gel

After obtaining aloe vera leaves from the Jiroft University Research Farm, the leaves were washed and the serrated edges were cut with a sharp knife. The upper layer of the leaf was removed lengthwise and the gel was carefully separated from the leaf. The gel pieces were homogenized in a blender and the resulting extract was filtered (Benítez et al. 2013). In this study, 100% concentration of aloe vera extract was used.

### 2.3 Preparation of Coating Formulations

Five coating formulations were prepared using 100% aloe vera gel as the coating matrix: Aloe vera gel alone, aloe vera gel containing 500 or 1000 ppm fennel essential oil, and aloe vera gel containing 500 or 1000 ppm lime peel essential oil. The essential oil concentrations were prepared by dilution in 1000 mL of aloe vera gel. Accordingly, 0.5 and 1.0 mL of each essential oil were added to obtain final concentrations of 500 and 1000 ppm, respectively. No emulsifier was used in the preparation of the coating formulations. The mixtures containing essential oils were homogenized for 10 min using a professional blender (ARSHIA Professional Blender, Model BL1401‐1658, Germany) to ensure uniform dispersion of the essential oils throughout the aloe vera gel.

### 2.4 Egg Samples and Experimental Design

A total of 300 fresh white‐shelled chicken eggs (Average weight: 62.39 ± 0.1) were obtained from a local retail center in Jiroft, Kerman Province, Iran. The eggs were visually inspected, and those with cracks, breakage, visible shell defects, or surface contamination were excluded. Intact eggs with relatively uniform size and appearance (with white shell color) were selected and randomly allocated to six experimental groups. The mean initial egg weight was 62.39 ± 0.43 g. The experimental groups were as follows: (1) uncoated control; (2) 100% aloe vera gel; (3) Aloe vera gel containing 500 ppm fennel essential oil; (4) Aloe vera gel containing 1000 ppm fennel essential oil; (5) Aloe vera gel containing 500 ppm lime peel essential oil; and (6) Aloe vera gel containing 1000 ppm lime peel essential oil. The eggs assigned to the coating treatments were immersed in the respective coating formulations for 1 min and then dried at room temperature. The immersion procedure was repeated for another 1 min, followed by complete drying at room temperature (Caner and Cansiz 2007). All coated and uncoated eggs were stored at 5°C for 30 days, and egg quality and microbial assessments were performed on days 1, 15, and 30 of storage. Figure 1 provides a summary of the treatments and experimental procedures.

**FIGURE 1:**
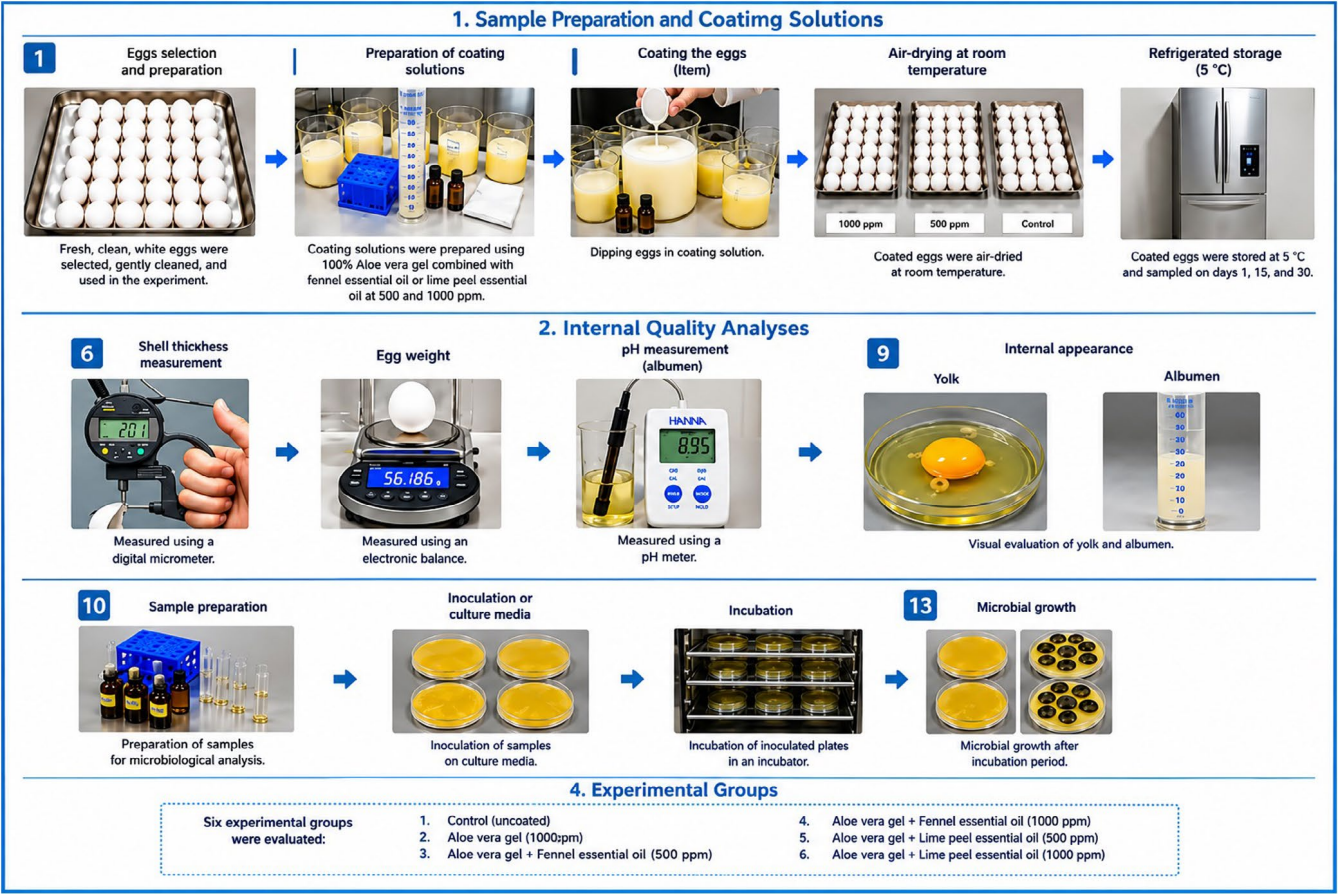
Schematic workflow in the Methods section.

### 2.5 Physical Properties

#### 2.5.1 Weight Loss

To measure weight loss, eggs were weighed individually at specified time intervals (1, 15, and 30 days of age) using a digital scale (A&D, GF‐3000, Japan) and then the percentage of weight loss was calculated using the following equation (Oliveira et al. 2023):

$$\begin{array}{c} \text{weight loss}\;\left(\%\right)=\left[\left(\text{initial weight}-\text{secondary weight}\right)/\text{initial weight}\right]\\ \times 100 \end{array}$$

#### 2.5.2 Determination of Haugh Unit

To determine the Haugh unit, the height of the thick part of the albumen was measured on days 1, 15, and 30 of storage using a digital caliper (GENERAL, USA; ±0.01). The resulting number was inserted into the following equation to determine the Haugh unit, where *W* is the egg weight (g) and *H* is the albumen height (mm) according to the original method described by Haugh (1937):

$$\text{Haugh unit}=100\;\log\;\left(H-1.7W^{0.37}+7.6\right)$$

#### 2.5.3 Shell Thickness

Shell thickness was measured using a micrometer with an accuracy of 0.001 mm on the first, fifteenth, and thirtieth days of storage. For this purpose, the eggs were first broken and their contents were removed. Then, the inner surface of the shell was washed with tap water and the shell membrane was completely separated. After the shell was dried at room temperature, its thickness was measured at five different points (top, middle, and bottom) with a micrometer. The average shell thickness (mm) was calculated from the average of the measurements taken at these five points (Suresh et al. 2015).

### 2.6 Chemical Tests

#### 2.6.1 Determination of pH of Albumen and Yolk

After complete separation of the yolk from the albumen, and homogenization with a glass rod, the pH of albumen and yolk was measured using a pH meter (Sartorius, Professional Meter PP‐50, Germany) calibrated with standard buffers (pH 4.01, 7.00, and 10.01) (Ezazi et al. 2021).

#### 2.6.2 Measurement of Albumen Foam Production

One hundred and fifty grams of albumen was weighed and stirred with an electric mixer at medium speed for 2 min and at high speed for 3 min until white, firm, and completely foamed. To determine the foam amount (foaming capacity), the volume of foam measured by a graduated beaker in ml was divided by the initial weight of albumen before stirring in g (Biladeau and Keener 2009).

#### 2.6.3 Microbial Test

To evaluate the microbial content of eggs, first the surface of the shell was disinfected with ethanol (70%). Then, under aseptic conditions, the shell was broken and the contents were emptied into a sterile beaker. The contents were homogenized, and 1 mL of the homogenized sample was mixed with 99 mL of sterile physiological saline. Serial decimal dilutions were then prepared up to 10^−2^. Subsequently, 100 μL of the prepared dilution series was spread onto Plate Count Agar (PCA) according to ISO 4833‐1:2013. The plates were incubated for 24 to 48 h at 37°C under aerobic conditions. After incubation, the colonies were counted and multiplied by the corresponding dilution factor. The results were calculated as colony‐forming units per gram of sample and reported as log_10_ CFU/g.

### 2.7 Statistical Analysis

The data obtained were statistically analyzed using IBM SPSS Statistics version 27.0 (IBM Corp., Armonk, NY, USA). The experiment was arranged as a factorial experiment in a completely randomized design. The effects of treatment, storage time, and their interaction were evaluated using factorial analysis of variance (ANOVA). Means were compared using Duncan's multiple range test at a significance level of *p* ≤ 0.05. All measurements were performed in triplicate.

## 3 Results

### 3.1 Weight Loss

The results of Table 1 show that with increasing storage time, the weight loss of all samples increased significantly (*p* ≤ 0.05). On the first day, no significant difference was observed between the weight loss of different samples (*p* > 0.05). At the end of the storage period, the highest weight loss was observed in the control treatment. The coated treatments showed significantly lower weight loss than the control; however, no significant differences were observed among the coated treatments (*p* > 0.05).

**TABLE 1:** Effect of edible aloe vera gel coatings enriched with fennel and lime peel essential oils on egg weight loss (%) during refrigerated storage.

Treatments	1 day	15 days	30 days
Uncoated	0.00Ca*	2.52B**a	4.81Aa
Coated with aloe vera gel (100%)	0.00Ca	1.15Bb	3.36Ab
Coated with aloe vera gel + 500 ppm fennel essential oil	0.00Ca	1.14Bb	3.35Ab
Coated with aloe vera gel + 1000 ppm fennel essential oil	0.00Ca	1.14Bb	3.35Ab
Coated with aloe vera gel + 500 ppm lime peel essential oil	0.00Ca	1.14Bb	3.35Ab
Coated with aloe vera gel + 1000 ppm lime peel essential oil	0.00Ca	1.14Bb	3.35Ab

* Different lowercase letters indicate a significant effect of treatment (*p* ≤ 0.05).

** Different capital letters indicate a significant effect of storage time (*p* ≤ 0.05).

### 3.2 Haugh Units

The results presented in Table 2 showed that the Haugh unit gradually decreased during storage in all treatments. At the end of the storage period, the highest Haugh unit values were observed in the treatments coated with aloe vera gel and 1000 ppm of fennel and lime peel essential oils, whereas the lowest Haugh unit value was observed in the uncoated control.

**TABLE 2:** Effect of edible aloe vera gel coatings enriched with fennel and lime peel essential oils on Haugh unit of eggs during refrigerated storage.

Treatments	1 day	15 days	30 days
Uncoated	76.21Ab*	66.80 B**d	54.17Cd
Coated with aloe vera gel (100%)	76.12Ab	69.20Bc	57.10Cc
Coated with aloe vera gel + 500 ppm fennel essential oil	76.28Ab	71.16Bbc	63.30Cab
Coated with aloe vera gel + 1000 ppm fennel essential oil	76.64Aab	73.76Aa	65.74Ba
Coated with aloe vera gel + 500 ppm lime peel essential oil	76.99Aa	71.98Bab	62.36Cb
Coated with aloe vera gel + 1000 ppm lime peel essential oil	76.98Aa	73.66Ba	65.40Ca

* Different lowercase letters indicate a significant effect of treatment (*p* ≤ 0.05).

** Different capital letters indicate a significant effect of storage time (*p* ≤ 0.05).

### 3.3 Shell Thickness

According to Table 3, the studied treatments and storage time did not have a significant effect on eggshell thickness (*p* > 0.05). Although a slight numerical reduction in shell thickness was observed during storage, this decrease was not statistically significant. The coated treatments also showed numerically greater shell thickness than the control.

**TABLE 3:** Effect of edible aloe vera gel coatings enriched with fennel and lime peel essential oils on eggshell thickness (mm) during refrigerated storage.

Treatments	1 day	15 days	30 days
Uncoated	0.410Aa*	0.384A**a	0.356Aa
Coated with aloe vera gel (100%)	0.414Aa	0.3860Aa	0.376Aa
Coated with aloe vera gel + 500 ppm fennel essential oil	0.412Aa	0.398Aa	0.384Aa
Coated with aloe vera gel + 1000 ppm fennel essential oil	0.414Aa	0.400Aa	0.394Aa
Coated with aloe vera gel + 500 ppm lime peel essential oil	0.412Aa	0.396Aa	0.382Aa
Coated with aloe vera gel + 1000 ppm lime peel essential oil	0.410Aa	0.396Aa	0.392Aa

* Different lowercase letters indicate a significant effect of treatment (*p* ≤ 0.05).

** Different capital letters indicate a significant effect of storage time (*p* ≤ 0.05).

### 3.4 Albumen pH

Table 4 shows that with increasing storage time, the pH of the samples increased and, in other words, the albumen of the studied samples became more alkaline during storage. At the end of the storage time, the highest albumen pH was observed in the control (without coating), and the lowest pH was observed in the treatments coated with aloe vera gel and 1000 ppm of fennel and lime peel essential oils. In general, the coated treatments had a lower pH than the control.

**TABLE 4:** Effect of edible aloe vera gel coatings enriched with fennel and lime peel essential oils on albumen pH during refrigerated storage.

Treatments	1 day	15 days	30 days
Uncoated	7.21Ca*	7.79B**a	8.26Aa
Coated with aloe vera gel (100%)	7.24Ca	7.59Bb	8.03Ab
Coated with aloe vera gel + 500 ppm fennel essential oil	7.20Ca	7.49Bcd	7.75Ac
Coated with aloe vera gel + 1000 ppm fennel essential oil	7.22Ca	7.44Be	7.60Ad
Coated with aloe vera gel + 500 ppm lime peel essential oil	7.21Ca	7.51Bc	7.76Ac
Coated with aloe vera gel + 1000 ppm lime peel essential oil	7.20Ca	7.46Bde	7.64Ad

* Different lowercase letters indicate a significant effect of treatment (*p* ≤ 0.05).

** Different capital letters indicate a significant effect of storage time (*p* ≤ 0.05).

### 3.5 Yolk pH

According to the results presented in Table 5, yolk pH increased with increasing storage time. At the end of storage, the highest yolk pH was observed in the uncoated control, whereas the lowest value was observed in the treatment coated with aloe vera gel containing 1000 ppm fennel essential oil, followed by the treatment containing 1000 ppm lime peel essential oil.

**TABLE 5:** Effect of edible aloe vera gel coatings enriched with fennel and lime peel essential oils on yolk pH during refrigerated storage.

Treatments	1 day	15 days	30 days
Uncoated	6.05Ca*	6.48B**a	6.91Aa
Coated with aloe vera gel (100%)	6.04Ca	6.37Bb	6.86Ab
Coated with aloe vera gel + 500 ppm fennel essential oil	6.03Ca	6.31Bc	6.65Ac
Coated with aloe vera gel + 1000 ppm fennel essential oil	6.05Ca	6.27 Bd	6.54Ae
Coated with aloe vera gel + 500 ppm lime peel essential oil	6.04Ca	6.31Bc	6.66Ac
Coated with aloe vera gel + 1000 ppm lime peel essential oil	6.03Ca	6.29Bcd	6.57Ad

* Different lowercase letters indicate a significant effect of treatment (*p* ≤ 0.05).

** Different capital letters indicate a significant effect of storage time (*p* ≤ 0.05).

### 3.6 Foam Production of Albumen

The results of the present study showed that the amount of foam produced by egg albumen decreased with increasing storage time (Table 6). This decrease was less pronounced in the coated samples than in the control. Although slight differences among coated treatments were observed on the first day, no significant differences were found among the coated treatments at the end of storage (*p* > 0.05).

**TABLE 6:** Effect of edible aloe vera gel coatings enriched with fennel and lime peel essential oils on albumen foaming capacity (mL/g of albumen) during refrigerated storage.

Treatments	1 day	15 days	30 days
Uncoated	8.35Aab*	7.87B**b	6.28Cb
Coated with aloe vera gel (100%)	8.40Aa	8.21Ba	7.51Ca
Coated with aloe vera gel + 500 ppm fennel essential oil	8.35Aab	8.21Ba	7.51Ca
Coated with aloe vera gel + 1000 ppm fennel essential oil	8.31Aab	8.19Ba	7.53Ca
Coated with aloe vera gel + 500 ppm lime peel essential oil	8.36Aab	8.22Ba	7.50Ca
Coated with aloe vera gel + 1000 ppm lime peel essential oil	8.30Ab	8.19Ba	7.50Ca

* Different lowercase letters indicate a significant effect of treatment (*p* ≤ 0.05).

** Different capital letters indicate a significant effect of storage time (*p* ≤ 0.05).

### 3.7 Total Microbial Count

With increasing storage time, the total microbial count of egg samples in all treatments increased (Table 7). Coated samples showed a smaller increase in total microbial count than the control. Addition of fennel and lime peel essential oils resulted in a decrease in microbial count. At the end of the storage, the lowest total microbial count was observed in samples coated with aloe vera gel and 1000 ppm of fennel and lime peel essential oils, and the highest total count was observed in uncoated samples (control).

**TABLE 7:** Effect of edible aloe vera gel coatings enriched with fennel and lime peel essential oils on total microbial count (log CFU/g) during refrigerated storage.

Treatments	1 day	15 days	30 days
Uncoated	2.79Ca*	3.93B**a	5.40Aa
Coated with aloe vera gel (100%)	2.77Ca	3.54Bb	4.67Ab
Coated with aloe vera gel + 500 ppm fennel essential oil	2.77Ca	3.25Bcd	4.35Ac
Coated with aloe vera gel + 1000 ppm fennel essential oil	2.75Ca	3.09Be	4.16Ad
Coated with aloe vera gel + 500 ppm lime peel essential oil	2.77Ca	3.27Bc	4.36Ac
Coated with aloe vera gel + 1000 ppm lime peel essential oil	2.75Ca	3.17Bde	4.17Ad

* Different lowercase letters indicate a significant effect of treatment (*p* ≤ 0.05).

** Different capital letters indicate a significant effect of storage time (*p* ≤ 0.05).

## 4 Discussion

### 4.1 Weight Loss

Evaporation of moisture and loss of carbon dioxide from the albumen through the porous shell to the external environment are the main reasons for egg weight loss, which is an important factor in evaluating egg quality (Xu et al. 2017). The increased weight loss of eggs during storage is mainly associated with water evaporation and carbon dioxide loss through eggshell pores (Faghei Shahrbabaki et al. 2025). The increased weight loss of eggs during storage may be due to the continuous evaporation of water and loss of carbon dioxide through the pores of the eggshell (Taha et al. 2019). As a result, the height of the air chamber increases with the decrease in the volume of the egg contents. In the present study, the use of different coatings on eggshells prevented weight loss during the storage period. It has been reported that coating prevents water from escaping through the pores of the shell and maintains the weight of egg samples (Khodadadi Baighout et al. 2023). This protective effect is mainly attributed to the formation of a semipermeable barrier on the eggshell surface, which reduces the diffusion of water vapor and carbon dioxide, thereby limiting moisture loss during storage. Similar improvements in the barrier properties of biopolymer coatings have also been reported by Yang et al. (2026).

In accordance with the present research, a study investigated the effect of aloe vera gel coating (100%) and different levels of chitosan on the weight loss of eggs stored at room temperature and reported that coated eggs had the lowest weight loss compared to uncoated eggs over a four‐week storage time (Kılınç et al. 2023). The positive effects of coatings on lower weight loss are likely due to the closure of eggshell pores, thereby maintaining the moisture and carbon dioxide levels of the eggs (Kılınç et al. 2023). da Silva Pires et al. (2021) reported that at the end of a six‐week storage time, eggs coated with rice protein concentrate and green propolis showed the lowest weight loss. In all treatments, weight loss gradually increased with increasing storage periods, and the highest weight loss was in the control (uncoated).

Due to their hydrophobic properties, plant essential oils can prevent moisture loss and thus reduce weight loss in eggs (Pires et al. 2020). Roudashtian et al. (2021) also reported that the use of carboxymethyl cellulose and tragacanth coatings containing clove extract prevents egg weight loss during storage by creating a strong barrier against the release of carbon dioxide gas. More recently, Faghei Shahrbabaki et al. (2025) reported that an optimized HPMC–nanoclay coating containing dill essential oil significantly reduced egg weight loss during storage compared with the uncoated control. In the study of Tajik et al. (2010), it was observed that the addition of oregano essential oil to chitosan and zein, compared to coated treatments without essential oil, prevented egg weight loss during 5 weeks of storage. However, in the present study, the use of essential oils in coating did not show a significant effect on weight loss of egg samples. This finding suggests that the moisture‐barrier properties of the coating were mainly provided by the aloe vera gel matrix. Under the conditions of the present study, the concentrations of fennel and lime peel essential oils were probably insufficient to further reduce water vapor transfer through the eggshell. Therefore, the main contribution of these essential oils was more likely related to their antimicrobial properties than to additional reduction in egg weight loss.

### 4.2 Haugh Units

The Haugh unit indicates the height of the thickest part of the albumen, and the higher its value, the higher the quality of the albumen. During storage, enzymes in the albumen hydrolyze the amino acid chains, which leads to disruption of the protein structure and the release of water. As a result, the albumen becomes watery and the viscosity of the dense albumen decreases (Oliveira and Oliveira 2013). The watery nature of the dense albumen causes a decrease in the Haugh unit value (Pires et al. 2020). As the pH increases during storage, the quality of the albumen deteriorates, followed by a decrease in the Haugh unit. The higher Haugh unit values observed in the coated eggs suggest that the coatings delayed albumen deterioration by reducing the loss of carbon dioxide through the eggshell pores, thereby slowing the increase in albumen pH and preserving the ovomucin network responsible for albumen viscosity (Rachtanapun et al. 2022). Several studies have shown that the Haugh index decreases with increasing storage time, and the use of coatings delays this decrease (Rachtanapun et al. 2022; Khademi Shurmasti et al. 2023).

Recent egg‐coating studies have likewise shown improved retention of the Haugh unit during storage (da Silva Pires et al. 2021; Pham et al. 2024; Faghei Shahrbabaki et al. 2025). Faghei Shahrbabaki et al. (2025) similarly reported that eggs coated with an optimized HPMC‐nanoclay film containing dill essential oil retained a higher Haugh unit than uncoated eggs during storage. The results of the present study are consistent with these findings, indicating that aloe vera gel, either alone or combined with fennel and lime peel essential oils, effectively preserved albumen quality during refrigerated storage by reducing changes associated with egg aging.

### 4.3 Shell Thickness

The present study showed that shell thickness was not significantly affected by either the coating treatments or storage time. This finding suggests that aloe vera gel and the incorporated essential oils mainly acted as protective surface barriers by reducing moisture and gas exchange rather than altering the mineralized structure of the eggshell. Similarly, Ehsan and Shurmasti (2022) reported that coating eggs with carboxymethyl cellulose containing nanoclay and marjoram extract ( *Origanum vulgare* L.) had no significant effect on shell thickness. In contrast, Senevirathne et al. (2022) observed greater measured shell thickness in beeswax‐coated eggs than in uncoated and coconut oil‐coated eggs, suggesting that changes in shell thickness may depend on the composition and physical characteristics of the coating material rather than changes in the eggshell itself.

### 4.4 Albumen pH

The pH value is one of the most important indicators for evaluating egg freshness and the measurement of egg albumen pH is usually used as a measure of the internal quality of eggs. Fresh egg albumen contains 0.5% carbon dioxide, which decreases during storage. The release of carbon dioxide from the egg leads to an increase in pH to about 8.5–9.6 (Caner and Cansiz 2007). In the present study, the lower albumen pH observed in coated eggs suggests that the coatings reduced carbon dioxide loss by acting as a semipermeable barrier, thereby delaying albumen alkalization (Khodadadi Baighout et al. 2023). Recent studies likewise show that edible coatings can limit the storage‐related increase in albumen pH by reducing gas exchange through the eggshell (Faghei Shahrbabaki et al. 2025; Favacho et al. 2025). Favacho et al. (2025) found that aloe vera coating initially limited the increase in albumen pH compared with uncoated eggs, although this protective effect diminished during prolonged storage. In a study, it was shown that the albumen pH of uncoated egg samples increased during storage at room temperature due to the exchange of gases, especially carbon dioxide, between the environment and the egg and the further alkalization of the albumen (Kim et al. 2009). An investigation into the effect of coating egg samples with wax, gelatin, and aloe vera gel on egg quality characteristics during storage at 30°C showed that uncoated samples showed a higher albumen pH than coated samples (Mudannayaka et al. 2016). In a study, the effect of coating quail eggs using 1% lime essential oil with green banana powder was investigated and it was reported that the lowest pH of the white was related to the coated treatments (Oliveira et al. 2023). In the study by Khodadadi Baighout et al. (2022), coating with black seed vegetable oil and rosemary extract was able to prevent excessive increase in the albumen pH of the egg by preventing the escape of moisture and carbon dioxide gas. These findings are consistent with the results of the present study, suggesting that aloe vera‐based coatings helped preserve albumen quality during storage.

### 4.5 Yolk pH

In addition to the Haugh unit and albumin pH, yolk pH can also be used as an indicator of egg freshness. The yolk pH in fresh eggs is around 6, but during egg storage, the pH gradually increases to 6.5 and above (Yuceer and Caner 2014). In a study, eggs coated with whey protein concentrate, corn zein and resin showed less changes in yolk pH than the control with increasing storage time. The yolk pH of eggs coated with resin was significantly lower than that of samples coated with whey protein concentrate or corn zein after 2 weeks of storage (Caner and Yüceer 2015). In the study of da Silva Pires et al. (2021), the changes in yolk pH during the storage time were different. After 6 weeks of storage, the yolk pH of uncoated samples increased from 6.13 to 6.99. The results showed that the use of rice protein concentrate and propolis coatings maintained the egg yolk pH, although there was no significant difference between the different coating treatments in yolk pH. Jahangir Esfahani et al. (2021) investigated the effect of coating eggs using UV‐C irradiated starch‐oleic acid biopolymer on egg quality and shelf life and reported that during the storage time, the yolk pH of the egg initially decreased (Weeks 1 to 3) and then increased (Weeks 3 to 7). At the end of the storage, the coated samples had a lower yolk pH than the control, but no significant difference was observed between the coated treatments. In the study of Pires et al. (2020) the yolk pH varied over the storage period. After 6 weeks of storage, the pH of the uncoated eggs increased from 6.22 to 7.05. From Week 3 to 5, the pH of the yolk in coated eggs with rice protein concentrate and different essential oils of tea tree, copaiba and thyme was lower than of the uncoated eggs. However, at Week 6, there was no difference among the pH of the yolk in control and any coated eggs. Few variations in pH of egg yolk were expected because the pH of the albumen increases during storage due to CO_2_ loss and migration of water from the albumen into the yolk during storage. Al Awwaly et al. (2024) showed that treatment without edible coating and edible coating (chitosan and casein edible with garlic essential oil) had a very significant effect on the pH of egg yolk. The longer the shelf life of eggs, the pH of yolk increases. This is due to the evaporation of CO_2_ causing the buffer system in egg white to decrease due to a dilution in the egg white and a permeation of H_2_O from the egg white to the yolk. Evaporation of CO_2_ causes the buffer system in egg whites to decrease due to a dilution in egg whites and an H_2_O permeation from egg whites to egg yolks.

### 4.6 Foam Production of Albumen

The ability of egg albumen to stir can be assessed by measuring the foam volume and foam stability (the amount of liquid released from the foam in a given time). Changes that occur in eggs during storage include albumen thinning, increasing pH, weakening and stretching of the vitelline membrane, and increasing yolk moisture content. In particular, ovomucin forms a film of insoluble materials between the liquid layer and the air bubble, thereby stabilizing the foam. Foam stability indicates the water‐holding capacity of the foam and is one way to determine the rate of liquid discharge from it (Kemps et al. 2010). In the present study, the higher foam production observed in coated eggs suggests that the coatings delayed the storage‐related changes in albumen, thereby helping to preserve its foaming properties. This finding can be explained by the ability of edible coatings to reduce moisture and carbon dioxide loss, which slows the increase in albumen pH and consequently preserves albumen functionality during storage (Caner and Yüceer 2015). In the study of Caner and Yüceer (2015), coating egg samples with whey protein concentrate, corn zein, and resin showed a significant interaction between coatings and storage time in terms of foam production. It was also found that with increasing storage time, the amount of foam produced in albumen decreases. Higher foam production indicates that more air is trapped in the egg albumen, which reduces the surface tension sufficiently to form a layer and surface film. Over time, these films gradually become thinner and break up (Kampf et al. 2003). During storage, the fluid is lost by the loss of water from the layer, resulting in the foam collapsing. Various coatings preserve the foam produced by the albumen as well as the whole egg, as they are an effective barrier against the loss of water and carbon dioxide and prevent changes in the pH of the egg albumen during storage (Caner and Yüceer 2015). In a study, it was found that the amount of albumin foam production during storage was higher in samples coated with black seed oil and rosemary extract than in the control. It was also found that the amount of foam production decreased during the storage period (Khodadadi Baighout et al. 2022). These findings are consistent with the results of the present study, confirming that coated eggs maintained higher foam production than the uncoated control throughout refrigerated storage.

### 4.7 Total Microbial Count

In the present study, coated eggs exhibited lower total microbial counts than the uncoated control throughout refrigerated storage, and the incorporation of fennel and lime peel essential oils further enhanced the antimicrobial effectiveness of aloe vera gel. This result suggests that the coating acted as both a physical barrier against microbial penetration and a carrier of natural antimicrobial compounds, thereby limiting microbial growth during storage. Various studies have demonstrated the antimicrobial effects of aloe vera gel, fennel, and lime peel essential oils. The antibacterial activity of aloe vera gel has been demonstrated against both Gram‐positive and Gram‐negative bacteria. The antimicrobial activity of aloe vera has been attributed to bioactive compounds such as anthraquinones, saponins, phenolic compounds, and organic acids, while the synergistic interaction among these compounds further enhances its antibacterial properties (Lawrence et al. 2009; Chelu et al. 2023). Arbab et al. (2021) also reported that aloe vera gel effectively inhibited the growth of common foodborne pathogens, including *Escherichia coli* , *Shigella*, *Salmonella*, and *Staphylococcus aureus* . Lime peel essential oil also possesses strong antimicrobial activity. Moosavy et al. (2017) reported that lemon peel essential oil significantly inhibited the growth of *Staphylococcus aureus* , mainly due to bioactive compounds such as limonene and *γ*‐terpinene, which disrupt bacterial cell membranes and interfere with essential cellular functions. Similarly, Azghar et al. (2023) demonstrated that lime peel essential oil exhibited broad‐spectrum antibacterial activity against both Gram‐positive and Gram‐negative bacteria.

The major antimicrobial constituents of fennel essential oil include trans‐anethole, fenchone, and methyl chavicol, which have been reported to inhibit the growth of a wide range of pathogenic microorganisms (Naaz et al. 2022). Di Napoli et al. (2022) further demonstrated that fennel essential oil inhibited both bacterial growth and biofilm formation, highlighting its potential as a natural antimicrobial agent in food preservation. The greater reduction in microbial counts observed in treatments containing essential oils compared with aloe vera gel alone may therefore be attributed to the complementary antimicrobial activities of aloe vera gel and the essential oils. Similar findings were reported by Ehsan and Shurmasti (2022), who observed that active edible coatings containing plant extracts significantly reduced microbial growth during egg storage. Overall, these results support the potential application of aloe vera gel enriched with fennel and lime peel essential oils as a natural coating for improving the microbial safety and extending the shelf life of eggs during refrigerated storage.

In the study by Khodadadi Baighout et al. (2022), the level of shell contamination after 6 weeks in the control sample reached 4.59 log CFU/g. In samples coated with black seed oil alone and in the presence of rosemary extract at 1%, 2%, and 3%, the level of contamination was estimated to be 4.32, 3.99, 3.27, and 2.30 log CFU/g, respectively. Due to the antimicrobial properties of the coating compounds, the microbial load of the shell decreased. The coating used was able to increase the shelf life of eggs by up to 6 weeks. The results of this study were consistent with the present study. Oliveira et al. (2023) reported that at the end of the storage period, quail eggs coated with green banana flour and Tahiti lemon essential oil had significantly lower total counts of aerobic mesophilic bacteria in the shell and egg contents compared to the control (uncoated sample).

## 5 Limitations

This study has some limitations. Only two concentrations of fennel and lime peel essential oils (500 and 1000 ppm) were evaluated, and the eggs were stored under one refrigerated condition (5°C) for 30 days. In addition, sensory properties and consumer acceptance of the coated eggs were not evaluated. Future studies could examine a wider range of essential oil concentrations and storage conditions and include sensory evaluation to better assess the practical application of these coatings.

## 6 Conclusion

In general, the results of the present study showed that coating egg samples with aloe vera gel containing fennel and lime peel essential oils improved egg quality by reducing moisture and carbon dioxide loss, thereby minimizing weight loss during refrigerated storage. Coatings containing fennel and lime peel essential oils also better maintained the Haugh unit and reduced the total microbial count compared with the uncoated control. These findings support the initial hypothesis that enriching aloe vera gel with fennel and lime peel essential oils enhances the preservative effectiveness of the coating by improving both egg quality and microbial stability during storage. Although these results were obtained under laboratory conditions, further studies are required to evaluate the industrial scalability, long‐term performance, and economic feasibility of this coating system. Overall, aloe vera gel containing 1000 ppm fennel essential oil or 1000 ppm lime peel essential oil can be considered a promising natural coating for extending egg shelf life and maintaining egg quality during refrigerated storage.

## Author Contributions

**Zahra Ranjbarinasab:** investigation, methodology, writing – original draft, formal analysis. **Fatemeh Shahdadi:** conceptualization, methodology, supervision, writing – original draft. **Maryam Yousefi:** methodology, formal analysis. **Zohreh Seyedimarghaki:** formal analysis, methodology, investigation. **Shima Omidi:** validation, formal analysis, writing – review and editing.

## Funding

The authors have nothing to report.

## Ethics Statement

This study did not involve human participants, clinical trials, or animal experimentation. The authors have nothing to report.

## Consent

This study did not involve human participants; therefore, informed consent was not required.

## Conflicts of Interest

The authors declare no conflicts of interest.

## Data Availability

The datasets generated and analyzed during the current study are available from the corresponding author upon reasonable request.
